# Efficacy of mobile-based educational intervention using Instructional Systems Design in promoting preventive behaviors for sexually transmitted infections among Iranian women: a randomized controlled trial

**DOI:** 10.1186/s12889-024-18002-1

**Published:** 2024-02-17

**Authors:** Afsaneh Karami Juyani, Fatemeh Zarei, Raziyeh Maasoumi

**Affiliations:** 1 Department of Health Education and Health Promotion, Faculty of Medical Sciences, Tarbiat Modares University, Tehran, Iran; 2 Department of Midwifery and Reproductive Health, School of Nursing and Midwifery, Tehran University of Medical Sciences, Tehran, Iran; 3 Nursing and Midwifery Care Research Center, School of Nursing and Midwifery, Tehran University of Medical Sciences, Tehran, Iran

**Keywords:** Sexually transmitted infections (STIs), Mobile health (mHealth), Women

## Abstract

**Background:**

Women who are sexually active are at risk of sexually transmitted infections (STIs), which can cause serious difficulties for their reproductive health. However, despite the high global burden of STIs, most infections are preventable with education for behavioral change. The purpose of this study is to investigate the Efficacy of Mobile-Based Educational Intervention Using Instructional Systems Design in Promoting Preventive Behaviors for Sexually Transmitted Infections among Iranian Women.

**Methods:**

This randomized controlled trial aimed at promoting preventive behaviors related to STIs in Iranian women with an educational intervention based on the Instructional Systems Design (ISD) in 2022. The participants in this study were recruited from a single center, specifically the Health House No. 3 located in District 11 of Tehran Municipality. Two instruments were used in the present study: a) a valid scale titled: “Four-Scale of STI Preventive Behaviors”, and b) a researcher-made Questionnaire titled: “Social perception affecting sexually transmitted infections (SOPESTI)”. These tools contain 8 demographic items and specific questions with a total of 68 five-point Likert scales. The intervention comprised three phases: a pre-test (baseline), a training program, and two follow-up assessments (4 and 12 weeks after the start of the training program). The experimental group received education through a mobile app, while the control group received no intervention. SPSS v.26 was used, with a significance level of *P* < 0.05. The chi-square test, Fisher’s exact test, independent t-tests, analysis of covariance (ANCOVA), and repeated measures ANOVA were used to analyze the data.

**Results:**

A total of 80 women, with a mean age of 36.524 ± 6.91 (experiment group) and 34.78 ± 8.20 (control group), respectively, participated in the trial. The study revealed a statistically significant difference in the mean score for eight domains, including STIs Knowledge, STIs Vulnerability, STIs Preventive Self-efficacy, STIs Prevention intentions, STIs Perceived social exclusion, STIs Perceived cognitive barriers, STIs Perceived social support, and STIs Perceived risks in the experiment group following the intervention compared to before the intervention (*p* < 0.05).

**Conclusion:**

The results of this study showed that a mobile-based educational intervention based on the ISD model had a significant effect on the preventive behaviors of STIs in Iranian women. These results highlight the potential benefit of mobile health in enhancing reproductive health.

**Trial registration:**

ClinicalTrials.gov IRCT20200602047638N1. Registered on 22 May 2021 with the IRCTID, V1.0. https://www.irct.ir/trial/55632

**Supplementary Information:**

The online version contains supplementary material available at 10.1186/s12889-024-18002-1.

## Introduction

Sexually transmitted infections (STIs) pose a considerable public health challenge, particularly in developing nations [1]. The World Health Organization (WHO) reports that globally, over 1 million cases of STIs are contracted each day [2]. In the United States, the estimated prevalence of eight common bacterial, viral, and parasitic STIs, including chlamydia, gonorrhea, and human papillomavirus, is 67.6 million, with an annual incidence of 26.2 million cases [2]. In the Eastern Mediterranean region, the WHO estimated approximately 26.4 million cases of four preventable STIs in 2008 [3].

### Status of STIs in Iran: statistics and prevention

Despite a global decrease in the number of newly acquired HIV infections, certain countries such as Iran are experiencing an upward trend in the incidence of HIV infection. By utilizing the UNAIDS spectrum and employing modeling techniques, it has been estimated that in the year 2019, the population of individuals living with HIV in Iran reached approximately 59,000 [4]. Furthermore, it is projected that on an annual basis, around 4,100 new infections and 2,500 AIDS-related deaths occur within the borders of the country. As indicated by the report generated by the Iranian national HIV registry system, a total of 38,966 individuals were officially diagnosed with HIV infection by the conclusion of the year 2018. Among this population, the majority consisted of males (83%), with an age range predominantly falling between 16 and 40 years (67.6%) [5]. Moreover, the number of HIV-infected individuals who succumbed to mortality due to any cause reached 15,845 by the end of 2018 [6]. Additionally, in the year 2018, a report from Iran revealed that out of a total of 1,080 patients, 155 individuals (14.3%) were diagnosed with C. trachomatis infection [7]. In 2022, the prevalence of combined syphilis in Iran was recorded to be 0.1%, with a prevalence rate of 0.4% in males and 0.6% in females. Furthermore, a review study conducted in 2022 demonstrated that the prevalence of HPV in women aged 18 to 59 years in Iran was reported to be 49.5% [8].

In adults can lead to various health complications such as cervicitis, urethritis, genital ulcers, pelvic inflammatory disease, chronic pelvic pain, ectopic pregnancy, infertility, neurological disorders, and cardiovascular disease. In infants, STIs can result in neonatal death, premature birth, blindness, and severe illnesses causing disabilities [9]. These complications disproportionately affect individuals of all ages, with significant consequences for women of reproductive age [10]. Biologically, women are more susceptible to these infections than men, making them more prone to experiencing complications [11, 12].

Research has shown that despite the significant global impact of these diseases, including economic [13], social, and psychological consequences [14], most of these infections can be prevented and treated [15]. Between 2010 and 2013, Iran’s third national program for AIDS and STIs had four approaches to tackle STIs: raising awareness, preventing transmission through sexual contact, providing treatment, and improving the epidemiological care system by managing data [16]. However, even with significant attempts to find straightforward solutions to decrease hazardous actions, altering behavior continues to be a difficult task [17]. There are several challenges in educating people about sexual health, particularly when it comes to STIs. Research has shown that in conservative sociocultural contexts, women often have limited knowledge about the signs and symptoms of STDs, as well as how to prevent, diagnose, and treat them. Misunderstandings and lack of knowledge can contribute to negative attitudes towards individuals with STIs, with infected women often facing more blame and condemnation than men [18].

Resistance to receiving sexual health education and limited access to effective resources can result in individuals obtaining incorrect information from virtual, and non-virtual sources [19, 20]. Additionally, discussing STIs in a conservative culture like Iran can be challenging and taboo [21, 22]. In these situations, providing a safe, supportive, and informative environment through mobile-based learning can be an effective strategy. The use of mobile apps allows for self-paced learning, which can appeal to younger generations [23]. Advances in information technology have led to the emergence of mobile health (mHealth), which involves the communication and delivery of health services via text messages, smartphone apps, websites, and social media. As a result, mHealth has become a highly accessible and adaptable approach to medical practice, particularly in low- and middle-income countries [24, 25]. With 70% of Iranians using smartphones [20], providing STI education through smartphone apps could be an alternative to more conservative methods of health education. The key consideration is the extent to which an educational program is based on learning technologies, regardless of its theoretical framework.

Research indicates that structured educational intervention programs are among the most effective strategies for promoting behavior change [26]. These programs provide individuals with the knowledge, information, and skills necessary to engage in healthy behaviors. Instructional design, which involves the systematic analysis, design, development, implementation, and evaluation of learning materials and activities, aims to shift the focus from a traditional teacher-centered approach to a learner-centered approach to teaching, thereby facilitating effective learning [27]. Given the above situation in Iran, this study aims to investigate the effectiveness of an educational intervention based on the ISD (Instructional Systems Design) model and using smartphone applications to promote STIs preventive behavior in women at risk. This study aimed to investigate the efficacy of mobile-based educational intervention using Instructional Systems Design to promote preventive behaviors for sexually transmitted infections among Iranian women.

## Methods

### Study design and participants

This study was a randomized controlled trial among Iranian married women of reproductive age referred to Tehran Municipality Health House, Iran in 2022. The participants in this study were recruited from a single center, specifically the Health House No. 3 located in District 11 of Tehran Municipality. The choice of this particular center was based on a report from the Women’s Empowerment Office affiliated with Tehran Municipality, which indicated a high prevalence of risky sexual behaviors in this district. Inclusion criteria included: 18 to 49 years old; possessing a smartphone with the capability to use mobile applications; self-declaration of not having cervical cancer and mental disorders; Self-declaration of not being addicted to drugs. To ensure accurate results, the study excluded women who did not use the app or did not use it fully, as well as those with a history of STIs or currently undergoing STI treatment. This study was registered with ClinicalTrials.gov IRCT20200602047638N1.

### Requirement

**Fig. 1:**
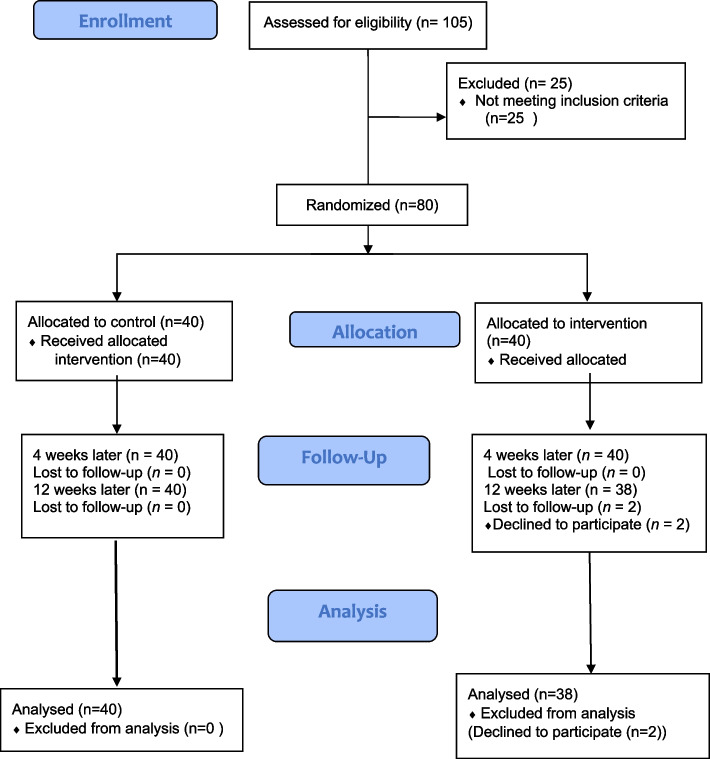
CONSORT flow diagram of the randomized controlled trials

In the initial phase, after coordinating with the Women’s Empowerment Office affiliated with Tehran Municipality, a list of 488 eligible women who had visited Health House No. 3 in District 11 over the past 6 months was obtained. The researcher then invited these women to participate in the study via digital platforms. Out of these, 105 women expressed interest in participating. The intervention was initially planned to be conducted in person at the Health House. However, due to the COVID-19 pandemic and the need to adhere to health protocols, along with the participants’ preference for non-physical participation, the study was conducted online. During the recruitment process, announcements were made on social networks, attracting 105 interested individuals. After initial screening, the number of participants was narrowed down to 80, as 25 individuals were unable to use the Android-based app required for the study. Each participant was assigned a unique research identification number and randomly allocated to either the experimental or control group using a simple randomization procedure. Participants were informed about the study before giving their informed consent. Due to the nature of the intervention, masking of participants and study staff was not possible. The randomization process and sample allocation are illustrated in the CONSORT flowchart (Fig. 1).

### Randomization

In this randomized controlled trial, participants were recruited after obtaining their informed consent and baseline data. A restricted randomized block design was used to allocate participants into either the experimental or control group, each participant being assigned a unique research identification number. The randomization process was conducted using a computer-generated plan available at Sealed Envelope (https://www.sealedenvelope.com/simplerandomiser/v1/lists), ensuring a 1:1 ratio between the two study arms. The experimental group, denoted by the letter A, underwent a 4-week training program. A total of ten individuals who volunteered for the study were randomly assigned to either the experimental or control group using a 4-block method. This method involves creating blocks of four participants each and randomly assigning each block to either group, ensuring a balanced distribution of participants.

The control group, denoted by the letter B, did not receive formal training. Due to the nature of the educational intervention and the allocation ratio, it was not possible to blind participants and study staff to group assignments. The random assignment process continued until the desired sample size was attained. The effectiveness of the intervention was evaluated at two post-test intervals: 4 and 12 weeks after the intervention. The total duration of the intervention, from baseline to the second post-test, was 16 weeks.

### Sample size

To achieve a 95% confidence level and 80% test power (calculated using G-Power software), the required sample size was determined. The high Cohen’s effect size on each variable in the experimental group compared to the control group (E. S = 0.7) was taken into consideration. After substituting the values in the formula, the minimum sample size in each group was calculated to be 34 people. However, with an estimated 15% dropout rate, the number of people in each group was increased to 40. Here is the formula used:

$$\begin{array}{l}{\text{n}}=2\times ({\text{z}}1-2+{\text{z}}1-\upbeta )2{\text{E}}.\mathrm{ S}2=2\times (1.96+0.84)2(0.7)2\approx 34\\ {\text{z}}0.975=1.96\,\,\,\,\,\,\,\,\,\,\,\,\,\,\,\,\,\,\,\,\,\mathrm{ z}0.8=0.84\end{array}$$

### Content creation for mobile application using instructional system design (ISD)

In this study, we designed a smartphone application to promote preventive behaviors related to STIs among women. The application was developed to provide accurate STI knowledge and enhance STI prevention skills. To guide the development process, we utilized Dick and Carey’s (2014) instructional-system design (ISD) model, which served as a framework for organizing all the components and achieving the desired goals [28] (Table 1). The ISD model encompasses ten steps, including identifying instructional goals, conducting instructional analysis, identifying learners and contexts, writing performance objectives, developing assessment instruments, devising instructional strategies, creating instructional materials, designing and conducting formative evaluation, designing and conducting summative evaluation, and revising instruction. In this paper, we present a detailed account of the entire developmental process of the smartphone application, elucidating each of the ten stages. Throughout the development process, feedback and input were sought from a panel of experts, consisting of a nurse, psychologist, medical doctor specializing in infectious diseases, sexologist, and health education program specialist. Furthermore, an expert from an information technology company and a UI/UX designer contributed to the creation of a customized smartphone application tailored specifically for women.

**Table 1:** Domains, instructional goals, subordinate skills, performance objectives and educational contents

**Domains**	**Instructional Goals**	**Subordinate Skills**	**Performance Objectives**	**Educational Contents**
**STIs Knowledge**	Women have STIs knowledge	Women can distinguish various types of STIs	* Women can describe symptoms and complications of syphilis, gonorrhea, chlamydia, genital herpes, genital warts, and AIDS. * Women can describe the treatment methods for syphilis, gonorrhea, chlamydia, genital herpes, genital warts, and AIDS. * Women can describe diagnostic methods for syphilis, gonorrhea, chlamydia, genital herpes, genital warts, and AIDS.	* STIs types, STIs complications and STIs symptoms * Diagnostic and treatment methods for STIs * STIs-infection routes * STIs-risk behaviors
		Women can explain STIs routes	* Women can describe STIs routes and characteristics for each disease. * Women can describe the behavioral risks of STIs	
**STIs Vulnerability**	Women perceive STIs vulnerability	Women can explain personal STIs vulnerability	* Women can describe the relationship between STIs and sexual intercourse	* The relationship between Women and STIs * Physical hazards, when infected with STIs
		Women can explain the harm of STIs	* Women can describe the harmful risks to genital health	
**STIs Preventive Self-efficacy**	Women have self-efficacy in STIs prevention	Women feel assured about how to use condoms Women feel self-assured that they know STIs risk factors	* Women can describe how to use condoms * Women can describe the advantages of condom use * Women can describe STIs risk factors * Women can describe STI-prevention methods	* Demonstration of condom use * Advantages of condom use * STI-related risk factors * Coping skills, when infected with STIs
		Women feel self-assured of their knowledge of STIs coping skills	* Adolescents can describe STI coping skills if they are infected with an STI	
**STIs Prevention intentions**	Women have STIs prevention intention	Women intend to use condoms to prevent STIs	* Women can describe that they will always use condoms to prevent STIs	* Condom use and sexual intercourse * Not having multiple sexual partners
		Women feel self-assured that they will not perform any STIs-risk behavior	* Women can describe that they will have sex with only one person to prevent STIs * Women can describe that they will not have sex with a person who is suspected of having an STIs * Women can describe that they will not have sex with a person with STIs	
**STIs Perceived social exclusion**	Women do not have STIs social exclusion	Women can explain STIs social exclusion	* Women can describe the relationship between STIs and social exclusion	* The relationship between STIs and social exclusion * Individual and social consequences of social exclusion
		Women can explain the effects of STIs social exclusion	* Women can describe the consequences of STIs social exclusion	
		Women can understand the effects of social exclusion caused by STIs	* Women can understand well the consequences of social exclusion caused by sexually transmitted diseases	
**STIs Perceived social support**	Women have STIs social support	Women can perceive social support related to STIs	Women can well understand the benefits of counseling and Getting information about STIs.	* Getting information about STIs through friends, doctors, midwives and healthcare staff * Getting information about STIs through books, magazines, media and the internet
**STIs Perceived cognitive barriers(fear)**	Women perceive STI cognitive Barriers	Women know the cognitive barriers associated with STIs	Women know that fear, embarrassment, guilt, etc. prevent preventive measures regarding sexually transmitted infections	* Types of cognitive barriers associated with STIs * Reduce or eliminate cognitive barriers associated with STIs
**STIs Perceived risks**	Women have STIs Perceived risk	Women understand being at risk	Women feel themselves at risk of STIs	* Ways of transmission of STIs

#### Identify instructional goals

The initial phase of the study involved determining the learning outcomes achievable through the educational process [28]. These outcomes were established based on need assessments, which involved analyzing the existing situation and identifying areas where improvement was necessary. To assess the current status of STI education among Iranian women, we employed both textual (through an extensive literature review) and contextual (through qualitative research) approaches. Through this systematic analysis, several issues in STI education were identified, including an excessive focus on AIDS, inadequate coverage of STI treatment and symptoms, and insufficient emphasis on STI prevention skills. To determine the desired content for STI education, we analyzed guidelines, educational standards, and content published by reputable sources such as the WHO, Centers for Disease Control and Prevention (CDC), and Iran’s Ministry of Health [3, 16, 17, 29]. Finally, we defined appropriate STI prevention content for eight domains STIs Knowledge, STIs Vulnerability, STIs Preventive Self-efficacy, STIs Prevention intentions, STIs Perceived social exclusion, STIs Perceived cognitive barriers, STIs Perceived social support, and STIs Perceived risks. Consequently, we determined instructional goals for women on STI-prevention intention to improve preventive behaviors related to STIs.

#### Conduct instructional analysis

At this phase, learners’ actions were guided by a hierarchical sequence of stages, once they had achieved the instructional objectives [28]. The instructional objectives encompassed two main steps: firstly, identifying the learning domains. This initial step involved categorizing the educational content into the eight specified domains, namely STIs Knowledge, STIs Vulnerability, STIs Preventive Self-efficacy, STIs Prevention intentions, STIs Perceived social exclusion, STIs Perceived cognitive barriers, STIs Perceived social support, and STIs Perceived risks. Secondly, specific objectives were established for each domain, leading to the formulation of an instructional goal for each domain.

#### Analyze learners and contexts

During this stage, we examined the learning experience, preferences, characteristics, and contexts of individuals about their understanding of STIs [28]. For Iranian women’s STI learning experience, currently, sex education is not common and no STI education is delivered in Health Care Centers (HCC), Municipality Health Houses, and even in national media. So far, when women refer to HCC to receive health care services and midwifery consultants the health care providers as educators teach STIs in face-to-face description most of the time without using relevant photos. Taking into account women’s preferences in educational formats, we opted for engaging mediums such as cartoons, animation, games, podcasts, and videos, instead of traditional descriptive approaches [30, 31]. Furthermore, considering the sensitive nature of STI education, an individualized educational approach was deemed advantageous, even within a conservative society like Iran [32, 33]. Hence, we devised a smartphone application-based educational method that integrated various multimedia elements including textual narratives, short films, motion graphics, games, podcasts, and images into a unified platform.

#### Write performance objectives

Upon completion of the educational program, learners are expected to develop a comprehensive action plan based on the knowledge acquired, in alignment with the established performance objectives [28]. We hypothesized that after receiving STI education, there would be observable improvements and changes in STIs knowledge, STIs vulnerability, STIs preventive self-efficacy, STIs prevention intentions, STIs perceived social exclusion, STIs perceived cognitive barriers, STIs perceived social support, and STIs perceived risks among women. Each objective is outlined in detail in Table 1. Subsequently, performance objectives were formulated by considering instructional goals and subordinate skills within the eight domains. Furthermore, we ensured that the terminology used in the performance objectives was simplified and made understandable to women through expert content verification.

#### Develop assessment instruments

In this phase, an evaluation of instruments is carried out to assess the extent to which learners have achieved their goals [28]. Therefore, two tools were employed to evaluate women’s preventive actions against sexually transmitted infections (STIs). One of these tools was a validated scale called the “STI Four-Scale of Preventive Behaviors”, which utilized constructs from the Motivation Prevention Theory (PMT) [34]. Additionally, a researcher-created questionnaire known as the “Social Perception Sexually Transmitted Infections” (SOPESTI) [35] was utilized to assess the psycho-social aspects of preventive behaviors related to STIs.

#### Develop instructional strategy

During the instructional strategy phase, the educator selects the appropriate educational approach for learners to achieve their desired learning outcomes [28]. In this study, we devised the instructional strategy based on Keller’s (1987) Attention, Relevance, Confidence, and Satisfaction (ARCS) model [36]. Keller’s model offers a systematic framework for designing the motivational aspects of a learning environment, aiming to initiate and sustain learners’ academic motivation. The first element, attention, was addressed by incorporating animated educational content to capture the attention of adolescents. The second element, relevance, focused on increasing learners’ educational motivation by presenting STI prevalence rates among women of reproductive age and highlighting the signs and symptoms of STIs through engaging game formats within the educational content. The third element, confidence, aimed to enhance learners’ belief in their ability to achieve educational goals. To instill confidence, we provided indirect experiences of condom usage through narratives derived from real-life experiences of other women. Additionally, learners were offered the flexibility of learning through smartphones, enabling them to revisit topics as needed. Finally, to promote learners’ satisfaction and achievement, we informed them that upon completing all the content, they would be able to demonstrate their understanding through assessment measures.

#### Develop instructional materials

During this phase, instructors engaged in the development and selection of instructional materials, guided by the chosen instructional strategy [28]. In light of this, we decided to collaborate with an information technology company to create a smartphone application specifically tailored for educating Iranian women about STIs. The app’s name, HESTIA, is an acronym derived from the first letters of the words ‘Health’, ‘Education’, ‘Sexuality’, ‘Transmitted’, ‘Infection’, and ‘Application’ in English. In Persian, HESTIA means a woman committed to a married life. The app’s logo features orange and green colors to symbolize a better future for all women and girls and health, respectively. HESTIA application was an Android-based application developed using the JAVA programming language by a team of designers who created its technical structure, UI, and UX.

#### Design and conduct formative evaluations of instruction

In the stage of formative evaluation, finding problems with education programs and assessment activities should be performed to improve the quality of the program [28]. Consequently, the educational material was disseminated to a minimum of 20 women who were not part of the research team, and the content’s validity was evaluated in terms of its relevance, comprehensibility, simplicity, and appeal. For this purpose, a questionnaire containing 10 questions along with the application link was sent to women through WhatsApp. Finally, after collecting the questionnaires, women’s opinions about the application were analyzed and the results obtained were used in the next stage.

#### Design and conduct summative evaluations

Summative evaluation is the stage of evaluation of the educational program of the whole [28]. In this stage, the educators reviewed the content and simplified it into messages, stories, short films, motion graphics, games, and podcasts. Finally, after modifications based on this feedback and obtaining partial satisfaction from 20 users, the final version of the program was published for educational intervention.

#### Final evaluation

The final evaluation based on the tenth step of the ISD model through the assessment instruments has been done through the Intervention step of this trial. A total of three time points were collected for both groups between April 9 to May 9, 2021: baseline (pre-test), 4 weeks following the completion of the intervention (post-test 1), and 12 weeks after the completion of the intervention (post-test 2). Details were explained in the Implementation of the STI education program.

##### Intervention: implementation of STI education program for women

Both the experimental and control groups were given an online pre-test questionnaire via WhatsApp or email. The experimental group was then introduced to a 4-week training program via the HESTIA app, with tutorial videos sent for installation and usage guidance. Post-installation, participants could contact researchers for assistance. The effectiveness of the intervention was evaluated using the STIs Four-Scale of Preventive Behaviors instrument and the SOPESTI questionnaire at two post-test intervals: 4 and 12 weeks after the intervention. The total duration of the intervention, from baseline to post-test 2, was 16 weeks. While the control group did not receive formal training, they were not isolated from external information sources. After the second post-test, they were also given access to the HESTIA app. It’s important to note that no special STI-related services were provided during the intervention. However, participants were offered a voucher for a free STI examination at a midwifery clinic as a token of appreciation.

### Instruments

To assess the preventive behaviors of women towards STIs, two instruments were used: a validated scale based on Motivation Prevention Theory (PMT) constructs titled “Four-Scale of STI Preventive Behaviors” [34], as well as a researcher-made questionnaire titled “Social Perception Sexually Transmitted Infections” (SOPESTI) [35] used to measure psycho-social aspect preventive behaviors in STI.

#### Four-Scale of STI Preventive Behaviors

STI Four-Scale of Preventive Behaviors is the Persian version [34] of a Korean tool for assessing preventive behaviors of STIs based on PMT. Four-Scale of STI Preventive Behaviors consisted of two sections: a) demographic items, and b) main items. The demographic section includes 8 items including age, education, occupation, economic status, marital status, age of marriage (duration of marriage), age of sexual intercourse, history of temporary marriage, and history of STDs. The main section consists of 40 items related to four dimensions, including STIs knowledge (28 Items), STIs vulnerability (4 Items), STIs Preventive self-efficacy (4 Items), and STIs Prevention intentions (4 Items). STIs Knowledge was scored with a range of 3 options: correct (2 points), don’t know (1 point), incorrect (0 points). The Likert scale was used to measure three additional dimensions, with the scoring ranging from 1 (strongly disagree) to 5 (strongly agree). In this tool, there is no special score or cutting level. There is no cut-off point for this tool. The total score of the tool is the sum of the scores of the four dimensions. The higher the score the greater the preventive STI behavior. Validity and reliability: The Content Validity Rate (CVR) and Content Validity Index (CVI) were computed and yielded a range of 0.56 to 1.00 and 0.83 to 1.00, respectively, indicating satisfactory content validity. To assess the reliability of the four items comprising the scale, Cronbach’s alpha and intraclass correlation coefficients were calculated, demonstrating values ranging from 0.660 to 0.850 and from 0.846 to 0.977, respectively.

#### Questionnaire of social perception affecting sexually transmitted infections (SOPESTI)

The SOPESTI is a research-made questionnaire for assessing social perceptions related to preventive behaviors in STIs. SOPESTI consists of 28 items related to social perceptions of STIs, including STIs Perceived social exclusion (7 Items), STIs Perceived cognitive barriers (fear) (7 Items), STIs Perceived social support (5 Items), and STIs Perceived risks (9 Items) that were measured on a Likert scale (very agree with a score of 5, agree: 4, no idea: 3, disagree: 2, and strongly disagree: 1). The impact score of all items was above 1.5, indicating that they were important and relevant. The calculated CVR and CVI were between 0.72 and 0.97 and 0.89 and 0.96, respectively, indicating good validity. The Cronbach’s alpha for each domain was as follows: STIs Perceived social exclusion (0.72), STIs Perceived cognitive barriers (fear) (0.71), STIs Perceived social support (0.75), and STIs Perceived risks (0.73), and the ICC ranges from 0.66 to 0.85, indicating good reliability.

### Data analysis

The type of analysis used in this study is Intention-To-Treat (ITT). In ITT analysis, all randomized participants are included in the analysis, regardless of whether they completed the intervention or adhered to the protocol [37]. Statistical analysis was performed using IBM SPSS Statistics Vr26. To assess the comparability of the two groups in terms of socio-demographic characteristics, the Chi-square test, Fisher’s exact test, and independent t-test were employed. To examine the differences in mean scores for women’s STIs Knowledge, STIs Vulnerability, STIs Preventive Self-efficacy, STIs Prevention intentions, STIs Perceived social exclusion, STIs Perceived cognitive barriers (fear), STIs Perceived social support, and STIs Perceived risks between the two groups before and after the intervention, independent t-tests and analysis of covariance (ANCOVA) were utilized. Furthermore, repeated measures ANOVA was conducted to compare the means of the eight domains within each group before and after the intervention. Post hoc analysis was performed using Bonferroni correction. A significance level of less than 0.05 (*P* < 0.05) was considered for all statistical tests. The analysis type in the CONSORT table had been done with a description of the statistical methods.

### Ethical considerations

The study protocol received approval from the Ethics Committee of Tarbiat Modares University, Tehran, Iran (code number: IR.MODARES.REC. 2020.039), which is the institution where the study was carried out. Before their involvement, all participants were provided with comprehensive written and verbal information regarding the study’s design, objectives, and potential benefits. Written informed consent was obtained from each participant, and they were assured that their personal information would remain confidential and anonymous throughout the study. Furthermore, the study materials, including questionnaires and informed consent forms, were approved by the university’s ethics committee.

## Result

### Socio-demographic characteristics

In this randomized controlled trial, a total of 80 women, ranging in age from 18 to 49 years, were enrolled. Among the 40 women assigned to the intervention group, two opted to discontinue their participation after a month. Consequently, the data analysis was conducted with a sample size of 78 women, comprising 40 women from the control group and 38 from the intervention group (Table 2).

**Table 2:** Comparison of socio-demographic characteristics of the participants between the two groups

**Demographic Variables**	N (%)			Test result
	Experiment (*n* = 38)	Control (*n* = 40)	Total (*n* = 78)	
**Women’s age (years)**				*p = 0.399
≤ 25	4 (10.5)	5 (12.5)	9 (11.53)	
26–35	13 (34.2)	18 (45)	31 (39.74)	
36–45	18 (47.4)	10 (25)	28 (35.89)	
≥ 46	3 (7.9)	7 (17.5)	10 (12.82)	
SD ± Mean	6.91 ± 36.24	8.20 ± 34.78	0.863 ± 35.09	
Minimum–Maximum	23–49	20–49	20–49	
**Age of starting sex(years)**				*p = 0.82
≤ 19	5 (13.15)	5 (12.5)	9 (11.53)	
20–29	24 (63.15)	28 (70)	52 (66.66)	
30–39	9 (23.70)	7 (17.5)	16 (20.5)	
SD ± Mean	4.94 ± 25.34	5.11 ± 24.61	0.534 ± 24.47	
Minimum–Maximum	14–36	17–35	14–36	
**Women’s marital status**				**p = 0.719
Permanent marriage	26 (68.4)	25 (62.5)	51 (65.38)	
Temporary marriage	5 (13.2)	8 (20)	13 (16.66)	
Divorced	7 (18.4)	7 (17.5)	14 (17.94)	
**Education level of women**				***p = 0.654
High school	3 (7.9)	6 (15)	9 (11.53)	
Diploma	9 (23.7)	8 (20)	17 (21.79)	
Academic	26 (68.4)	26 (65)	52 (66.66)	
**Employment status**				**p = 0.913
Housewife	11 (28.9)	14 (35)	25 (32.05)	
Student	6 (15.8)	7 (17.5)	13 (16.66)	
Employee	15 (39.15)	13 (32.5)	28 (35.89)	
Freelancer	6 (15.8)	6 (15)	12 (15.38)	
**Economic status**				***p = 0.942
Very favorable	2 (5.3)	1 (2.5)	3 (3.84)	
Favorable	24 (63.2)	25 (62.5)	49 (62.82)	
Unfavorable	10 (26.3)	12 (30)	22 (28.20)	
Very Unfavorable	2 (5.3)	2 (5)	4 (5.12)	
**Temporary marriage history**				**p = 0.530
Yes	9 (23.7)	12 (30)	21 (26.92)	
No	29 (76.3)	28 (70)	57 (73.07)	
**History of STIs**				**p = 0.211
Yes	6 (15.8)	11 (27.5)	17 (21.79)	
No	32 (84.2)	29 (72.5)	61 (78.20)	

^*^Independent t-test

^**^Chi-square test

^***^Fisher’s exact test

### The primary outcomes: eight measured variables

The primary outcome of this study was to determine and compare the effects of an educational intervention, designed based on the ISD model, on women’s knowledge of STIs, their perceived vulnerability, self-efficacy in prevention, prevention intentions, perceived social exclusion, perceived social support, cognitive barriers (fear), and perceived risks. These outcomes were measured among women in both the target and control groups before the intervention, and then again 4 weeks and 12 weeks post-intervention.

### STIs knowledge

Before the training, an independent t-test revealed a statistically significant difference in women’s knowledge about six diseases (AIDS, Chlamydia, Syphilis, Gonorrhea, Genital Herpes, and Genital Warts) between the test and control groups (*p* = 0.041). However, covariance analysis showed that this difference became statistically significant 1 month (*p* < 0.001) and 3 months (*p* < 0.001) post-intervention, with the test group scoring significantly higher on average than the control group. Repeated measures ANOVA indicated a statistically significant difference in the test group’s average knowledge score at least once (*p* < 0.001). Pairwise Bonferroni comparison revealed that this score was significantly higher 1 month (*p* < 0.001) and 3 months (*p* < 0.001) post-intervention compared to pre-intervention scores. Interestingly, the comparison of the average knowledge score between 1 month and 3 months after the intervention was statistically significant (*p* < 0.001). In contrast, the control group’s average knowledge score remained unchanged throughout the study (Table 3).

**Table 3:** The average scores of the eight domains before and after the educational intervention in the experiment and control groups

Variable	Pre-test	1 Month after intervention	3 Months after intervention	Test result
	**Mean ± SD**	**Mean ± SD**	**Mean ± SD**	
STIs Knowledge				
Experiment	28.36 ± 6.04	47.64 ± 3.50	41.18 ± 4.97	***p < 0.001
Control	25.95 ± 3.90	26.77 ± 4.09	26.10 ± 6.88	***p = 0.612
Test result	*p = 0.041	**p < 0.001 ${{\varvec{\eta}}}^{2}$** =** **0.880**	**p < 0.001 ${{\varvec{\eta}}}^{2}$** =** **0.595**	
STIs Vulnerability				
Experiment	12.26 ± 2.55	15.39 ± 2.44	15 ± 2.10	***p < 0.001
Control	11.50 ± 2.94	11.95 ± 2.56	11.57 ± 2.57	***p = 0.659
Test result	*p = 0.22	**p < 0.001 ${{\varvec{\eta}}}^{2}$** =** **0.317**	**p < 0.001 ${{\varvec{\eta}}}^{2}$** =** **0.339**	
STIs Preventive Self-efficacy				
Experiment	10.05 ± 3.12	16.55 ± 1.46	16.15 ± 2	***p < 0.001
Control	9.60 ± 1.94	10.65 ± 1.79	9.75 ± 2.59	***p = 0.033
Test result	*p = 0.44	**p < 0.001 ${{\varvec{\eta}}}^{2}$** =** **0.781**	**p < 0.001 ${{\varvec{\eta}}}^{2}$** =** **0.658**	
STIs Prevention intentions				
Experiment	12.05 ± 3.48	17.10 ± 2.05	16.86 ± 1.98	***p < 0.001
Control	10.85 ± 3.12	11.30 ± 2.37	10.02 ± 2.40	***p = 0.90
Test result	*p = 0.11	**p < 0.001 ${{\varvec{\eta}}}^{2}$** =** **0.651**	**p < 0.001 ${{\varvec{\eta}}}^{2}$** =** **0.703**	
STIs Perceived social exclusion				
Experiment	24.315 ± 4.574	20.631 ± 5.354	20.842 ± 5.902	***p = 0.001
Control	25.400 ± 4.573	25.100 ± 5.006	25.125 ± 3.837	***p = 0.857
Test result	*p = 0.299	**p < 0.001 ${{\varvec{\eta}}}^{2}$** =** **0.155**	**p < 0.001 ${{\varvec{\eta}}}^{2}$** =** **0.150**	
STIs Perceived cognitive barriers (fear)				
Experiment	23.078 ± 6.006	13.236 ± 4.812	12.105 ± 2.938	***p < 0.001
Control	21.625 ± 4.902	21.525 ± 5.058	22.725 ± 4.950	***p = 0.325
Test result	*p = 0.244	**p < 0.001 ${{\varvec{\eta}}}^{2}$** =** **0.513**	**p < 0.001 ${{\varvec{\eta}}}^{2}$** =** **0.638**	
STIs Perceived social support				
Experiment	14.50 ± 3.85	19.34 ± 4.02	19.05 ± 4.50	***p < 0.001
Control	13.12 ± 3.68	13.20 ± 4.85	14.35 ± 5.87	***p = 0.426
Test result	*p = 0.11	**p < 0.001 ${{\varvec{\eta}}}^{2}$** =** **0.304**	**p < 0.001 ${{\varvec{\eta}}}^{2}$** =** **0.158**	
STIs Perceived risks				
Experiment	27.31 ± 5.34	38.65 ± 4.27	36.84 ± 4.64	***p < 0.001
Control	26.62 ± 4.37	27.05 ± 5.01	25.94 ± 6.46	***p = 0.489
Test result	*p = 0.53	**p < 0.001 ${{\varvec{\eta}}}^{2}$** =** **0.654**	**P < 0.001 ${{\varvec{\eta}}}^{2}$** = 0.483**	

^*^Independent T-test

^**^Analysis of covariance (ANCOVA)

^***^Repeated measures ANOVA

### STIs vulnerability

Before the training, the perceived susceptibility of women to STIs, as determined by an independent t-test, did not exhibit a statistically significant difference between the test and control groups (*P* = 0.22). However, covariance analysis revealed that this difference became statistically significant in both groups 1 month (*P* < 0.001) and 3 months (*P* < 0.001) post-intervention, with the test group scoring significantly higher on average than the control group. Repeated measures ANOVA indicated a statistically significant difference in the test group’s average perceived vulnerability score at least once (*P* < 0.001). A pairwise Bonferroni comparison revealed that this score was significantly higher 1 month (*P* < 0.001) and 3 months (*P* < 0.001) post-intervention compared to pre-intervention scores. Notably, there was no statistically significant difference between the 1-month and 3-month post-intervention scores (*P* = 0.896). In contrast, the control group’s average perceived vulnerability score remained unchanged throughout the study (Table 3).

### STIs preventive self-efficacy

Before the training, an independent t-test revealed no statistically significant difference in women’s self-efficacy regarding STIs between the test and control groups (*p* = 0.44). However, covariance analysis showed that this difference became statistically significant 1 month (*p* < 0.001) and 3 months (*p* < 0.001) post-intervention, with the test group scoring significantly higher on average than the control group. Repeated measures ANOVA indicated a statistically significant difference in the test group’s average self-efficacy score at least once (*p* < 0.001). A pairwise Bonferroni comparison revealed that this score was significantly higher 1 month (*p* < 0.001) and 3 months (*p* < 0.001) post-intervention compared to pre-intervention scores. Interestingly, there was no statistically significant difference between the 1-month and 3-month post-intervention scores (*p* = 0.351). In contrast, repeated measures ANOVA showed a statistically significant difference in the control group’s average self-efficacy score at least once (*p* = 0.033). Bonferroni’s comparison revealed that the self-efficacy score 1-month post-intervention (*p* < 0.001) was significantly higher than the pre-intervention score. However, this difference was not significant at other times (refer to Table 3).

### STIs prevention intentions

Before the training, an independent t-test revealed no statistically significant difference in women’s intention to prevent STIs between the intervention and control groups (*p* = 0.11). However, covariance analysis showed that this difference became statistically significant 1 month (*p* < 0.001) and 3 months (*p* < 0.001) post-intervention, with the intervention group scoring significantly higher on average than the control group. Repeated measures ANOVA indicated a statistically significant difference in the test group’s average prevention intention score at least once (*p* < 0.001). A pairwise Bonferroni comparison revealed that this score was significantly higher 1 month (*p* < 0.001) and 3 months (*p* < 0.001) post-intervention compared to pre-intervention scores. Interestingly, there was no statistically significant difference between the 1-month and 3-month post-intervention scores. In contrast, the control group’s average prevention intention score remained unchanged throughout the study (Table 3).

### STIs perceived social exclusion

Before the training, an independent t-test revealed no statistically significant difference in women’s perceived social exclusion regarding STIs between the intervention and control groups (*p* = 0.299). However, covariance analysis showed that this difference became statistically significant 1 month (*p* < 0.001) and 3 months (*p* < 0.001) post-intervention, with the intervention group scoring significantly higher on average than the control group. Repeated measures ANOVA indicated a statistically significant difference in the test group’s average perceived social stigma score at least once (*p* < 0.001). Bonferroni’s two-way comparison revealed that this score was significantly lower 1 month (*p* = 0.003) and 3 months (*p* = 0.009) post-intervention compared to pre-intervention scores. Interestingly, there was no statistically significant difference between the 1-month and 3-month post-intervention scores. In contrast, the control group’s average perceived social stigma score remained unchanged throughout the study (Table 3).

### STIs perceived cognitive barriers(fear)

Before the training, an independent t-test revealed no statistically significant difference in women’s perceived cognitive barriers regarding STIs between the test and control groups (*p* = 0.244). However, covariance analysis showed that this difference became statistically significant 1 month (*p* < 0.001) and 3 months (*p* < 0.001) post-intervention, with the test group scoring significantly higher on average than the control group. Repeated measures ANOVA indicated a statistically significant difference in the test group’s average perceived cognitive barriers score at least once (*p* < 0.001). Bonferroni’s two-way comparison revealed that this score was significantly lower 1 month (*p* < 0.001) and 3 months (*p* < 0.001) post-intervention compared to pre-intervention scores. Interestingly, there was no statistically significant difference between the 1-month and 3-month post-intervention scores (*p* = 0.169). In contrast, the control group’s average perceived cognitive barriers score remained unchanged throughout the study (Table 3).

### STIs perceived social support

Before the training, an independent t-test revealed no statistically significant difference in women’s perceived social support regarding STIs between the test and control groups (*p* = 0.11). However, covariance analysis showed that this difference became statistically significant 1 month (*p* < 0.001) and 3 months (*p* < 0.001) post-intervention, with the test group scoring significantly higher on average than the control group. Repeated measures ANOVA indicated a statistically significant difference in the test group’s average perceived social support score at least once (*p* < 0.001). Pairwise Bonferroni comparison revealed that this score was significantly higher 1 month (*p* < 0.001) and 3 months (*p* < 0.001) post-intervention compared to pre-intervention scores. Interestingly, there was no statistically significant difference between the 1-month and 3-month post-intervention scores. In contrast, the control group’s average perceived social support score remained unchanged throughout the study (Table 3).

### STIs perceived risks

Before the training, an independent t-test revealed no statistically significant difference in women’s perceived risk of STIs between the test and control groups (*p* = 0.53). However, covariance analysis showed that this difference became statistically significant 1 month (*p* < 0.001) and 3 months (*p* < 0.001) post-intervention, with the test group scoring significantly higher on average than the control group. Repeated measures ANOVA indicated a statistically significant difference in the test group’s average perceived risk score at least once (*p* < 0.001). Bonferroni’s two-way comparison revealed that this score was significantly higher 1 month (*p* < 0.001) and 3 months (*p* < 0.001) post-intervention compared to pre-intervention scores. Interestingly, there was no statistically significant difference between the 1-month and 3-month post-intervention scores (*p* < 0.001). In contrast, the control group’s average perceived risk score remained unchanged throughout the study (Table 3).

#### The secondary outcome

The secondary outcome was to measure the impact of the educational intervention on women’s preventive actions towards STIs, such as using a condom, getting a pap-test, and undergoing a genital examination. However, due to the constraints imposed by the COVID-19 pandemic in Iran, these secondary outcomes were not evaluated as Health Care Centers were not permitted to be overloaded with additional services, such as genital examinations or pap test for research-based demands.

## Discussion

This study sought to evaluate the efficacy of an educational intervention grounded in the Instructional Systems Design (ISD) model in enhancing women’s preventive behaviors regarding sexually transmitted infections (STIs). The findings of the study reveal a statistically significant disparity in the average scores of women’s STI knowledge between the experiment group and the control group following the implementation of the educational intervention. This finding is supported by various other similar studies that have emphasized the significant role of digital health interventions based on innovative technologies such as smartphone applications and websites in increasing knowledge, improving attitudes, and promoting preventive behaviors against STIs [20, 38, 39]. Before the intervention, the experiment and control groups were homogeneous in all domains except for knowledge (*p* = 0.041). It is worth noting that women in the training group may have developed a greater interest in the study topic during the interval between joining the study and taking the pretest. This could have led to their acquisition of information about STIs and a higher level of knowledge in the experiment group compared to the control group before the pretest.

The educational intervention in this study was effective in increasing women’s STIs Vulnerability in the experiment group. In support of our findings, several other studies have shown that educational interventions based on applications can increase individuals’ Perceived vulnerability to STIs [20, 38–40].

In the study by Sutherland, Owusu, and et.al, a high level of STIs Vulnerability led to a reduction in risky sexual behaviors [39, 41]. However, some studies did not align with our results [42, 43]. For example, Zhang’s study showed no significant difference in the mean scores of students’ perceived vulnerability before and 6 months after the educational intervention [43]. It seems that the difference in target groups and the small number of items in Zhang’s vulnerability assessment tool, as well as the incomplete assessment of vulnerability, may be reasons for the discrepancy between these studies and our results. A notable point in many studies is that participants consider the probability of contracting STIs to be very low; in their view, only individuals with risky sexual behaviors and drug users are at risk of contracting STIs. This misunderstanding of vulnerability reduces individuals’ motivation and intention to prevent STIs. Furthermore, the findings of the present study stand in contrast to the results obtained by Soltani et al. [44] and Mohammadi et al. [45] concerning perceived severity. This discrepancy may be ascribed to variations in the gender composition of the target group, the quantity of training sessions conducted, or the cultural attributes inherent to the study population.

The educational intervention in this study was effective in increasing women’s preventive self-efficacy toward STIs in the training group. The results of a similar study are consistent with this finding [46, 47]. In line with the results of Leitch et al., high preventive self-efficacy and perceived sensitivity predict HPV vaccination intake and are effective in preventing risky sexual behaviors [48]. Boone et al. also confirm the impact of improving preventive self-efficacy on increasing sexual and reproductive health [49]. The results of our study present a divergence from the findings reported by McClendon et al. [50], particularly about the lack of notable alterations in the structure of self-efficacy. It is evident from our research that the pedagogical materials, which were meticulously designed to meet the educational needs of the experimental group, exerted a significant influence on the enhancement of their self-efficacy.

In this study, it was observed that the intention to prevent STIs significantly increased in the experiment group. Research indicates that this intention to prevent STIs plays a significant role in preventing STI infection as one of the important variables in determining behavior [20, 38, 49]. Furthermore, various studies [38, 39, 51] have shown that sexual transmission prevention educational programs can be significantly effective in increasing the intention to prevent STIs. For example, the results of the study by Rendina et alshowed that behavioral intention, as a predictive factor, contributes to an increase in the use of preventive methods such as condom use [49]. Sexual transmission prevention educational programs can be considered as a key component for preventive behaviors by increasing the intention to prevent STIs [51]. In the current study, an increase in knowledge was found to increase the intention to prevent risky behaviors. It seems that the educational content provided in the HESTIA app, which was designed proportionally to the needs of the audience, was able to bring about changes in this important area.

In this study, Perceived social exclusion related to STIs significantly decreased in the experiment group. Previous studies [48, 52–54] have emphasized that educational programs for the prevention of STIs can have a positive impact on Perceived social exclusion about STIs. The results of the study by Varas-Díaz et al. showed that participants in the intervention group had lower levels of Perceived social exclusion related to STIs compared to the control group [54]. Other studies have highlighted the positive role of family support and peer-based interventions in reducing perceived social exclusion by families [48, 55]. The results of the present study suggest that women had high levels of perceived social exclusion related to STIs before the intervention, The designed intervention successfully reduced the average score of perceived social exclusion related to STIs among women in the experiment group after the educational intervention.

The results of the study demonstrated that the designed intervention successfully reduced the average score of STIs Perceived cognitive barriers(fear) significantly after the educational intervention in the experiment group; Some of these study results are consistent with other research studies [55–57]. Both Sadeghi’s and Huang’s studies found a decrease in Perceived barriers among participants in the experimental group compared to the control group following the intervention [58]. However, the findings of this study contradict the results reported by Ghafari [59]. The differences in the perceived barriers to adopting preventative behaviors, including physical, material, psychological, and social barriers, and the different impacts of intervention programs on amending these barriers may have contributed to such inconsistencies. Changing incorrect beliefs and concepts about healthy behaviors can greatly assist in the prevention of STIs. it appears that even with high levels of perceived benefits, changing behavior will remain difficult until barriers to adopting healthy behaviors are addressed. Therefore, aligning these two constructs can significantly contribute to the emergence of healthy behaviors. According to Dillard, changing behavior is difficult until the perceived barriers are overcome [60].

The educational intervention designed in this study was effective in increasing perceived social support regarding STIs among the participants. Studies have shown the positive impact of social support on healthy behaviors [55–57, 61]. Social support is one of the constructs of social cognition theory, in which environmental factors (social and physical) directly affect individuals’ self-efficacy to engage in healthier behaviors. In this study, it appears that an increase in knowledge about STIs led to an increase in perceived social support (emotional and informational), which in turn reduced the in-group stigma and social stigma perceived by women. This result is consistent with those of previous studies [48, 53]. Studies have also identified family support as a key factor in reducing perceived stigma [15, 56, 61], increasing self-confidence [61] and also coping with the stress of HIV/AIDS, and improving quality of life [15, 60] in families with STIs.

Educational programs designed to increase the perceived risk of STIs among women were effective. Consistent with this study, other studies have shown that educational interventions based on theory are effective in increasing perceived risk [58, 62–65]. Also, the results of studies showed women with low educational levels had poorer knowledge of STIs and lower perceived risk of contracting them. Despite awareness of HIV cases in their communities, most women perceived their risk of contracting STIs as low, particularly those with limited education, and many argued that being monogamous, avoiding multiple sexual partners, and maintaining personal hygiene protected them.

### Strength and limitations

The study successfully developed and implemented an educational intervention to increase perceived risk related to STIs among women in Iran, using a mobile app as the delivery platform. However, the sample size was small, and only women with high digital literacy and access to a smartphone participated in the study.

To overcome these limitations, future studies could use a larger sample size, and study women with different demographic characteristics. The use of technology-based interventions should be taken into consideration in future studies, as they can be more effective in educating women about sexual health and increasing awareness of the risks associated with STIs. Moreover, there is a need to overcome the cultural taboo associated with the topic in Iran, and future studies could include different cultural values and beliefs to better understand the perspectives and behaviors of women in this regard. Finally, the COVID-19 pandemic has had a significant impact on the delivery and participation of the study, which is why future investigations should be conducted during more stable times to minimize any external effects.

### The clinical implication

The clinical implication of our study is the potential application of our educational intervention to improve the sexual health and well-being of women in Iran and other similar contexts. Our study shows that using a mobile app based on the ISD model can effectively increase the knowledge, awareness, and preventive behaviors of women regarding STIs, as well as reduce the social stigma and barriers associated with the topic. This implies that the intervention can be a valuable method for health professionals and organizations to educate and empower women to protect themselves and their partners from STIs, and to seek timely diagnosis and treatment if needed. The present intervention can also contribute to the prevention and control of STIs at the population level, by reducing the transmission and prevalence of these infections.

## Conclusion

In conclusion, the findings of this study demonstrate a significant improvement in women’s STIs Perceived social exclusion, STIs Perceived cognitive barriers (fear), STIs Perceived social support, and STIs Perceived risks, as well as their STIs Knowledge, STIs Vulnerability, STIs Preventive Self-efficacy, and STIs Prevention intentions. These results highlight the positive influence of an educational intervention that was specifically tailored to the needs of women and developed based on the ISD model, effectively promoting preventive behaviors related to STIs. Therefore, it is advisable to incorporate this type of education into sexual health programs targeting women in Iran, particularly those from low-income backgrounds or with limited access to formal education. Subsequent research endeavors should explore the impact of mobile app-based education on sexual health and preventive behaviors in greater depth.

### Supplementary Information

**Supplementary material 1.**

## Data Availability

The datasets utilized and/or analyzed in the present study can be obtained from the corresponding author upon reasonable request.

## Abbreviations

STIs: Sexually Transmitted Infections
STDs: Sexually Transmitted Diseases
ISD: Instructional System Design
RCT: Randomized controlled trial
CONSORT: Consolidated Standards of Reporting Trials. mHealth: Mobile health
