# Supplementary material for: Efficacy of mobile-based educational intervention using Instructional Systems Design in promoting preventive behaviors for sexually transmitted infections among Iranian women: a randomized controlled trial
Source: BMC Public Health. 2024 Feb 17;24:510. doi: 10.1186/s12889-024-18002-1 (PMC10874553; doi:10.1186/s12889-024-18002-1)
Supplement: Supplementary file 1 — Supplementary material 1. [file 12889_2024_18002_MOESM1_ESM.docx]

**Social Perceptions on Sexually Transmitted Infections (SOPESTI)**

**SECTION ONE: Tool description**

**Scaling**

**SOPESTI** is an acronym for Social Perceptions of Sexually Transmitted Infections, and it is a questionnaire used to assess Social Perceptions of Sexually Transmitted Infections. This tool is based on a conventional qualitative content analysis approach. Accordingly, four themes related to sexually transmitted infections (STIs), including the perceived social exclusion associated with STIs, the perceived risks associated with STIs, the perceived social support available for people with STIs, and the perceived cognitive barriers (fear) associated with STIs. A questionnaire was developed using 28 Likert five-point response items. The response options ranged from "completely agree" to "completely disagree".

**Scoring**

There is no cut-off point for this tool. STIs Perceived social exclusion scale was measured with 7 items with a 4-point Likert scale ranging from completely agree (4 points) to completely disagree (1 point). The total score for each item was 7-28. A higher score on the STIs Perceived Social Exclusion scale indicates a belief that the person experiences more social stigma. The scale of STIs Perceived cognitive barriers(fear)also included 7 items with a 4-point Likert scale ranging from completely agree (4 points) to completely disagree (1 point). STIs Perceived cognitive barriers(fear) The total score for each scale was 7-28. A higher score on the scale indicates the belief that a person experiences more cognitive barrier

**Interpretation**

Regarding the items designed in the section on perceived risk about STIs and STIs Perceived social support, the higher the score, the more preventive behaviors from STIs are, but regarding the items designed in the section on perceived social fear and STIs Perceived cognitive barriers(fear), conversely, the lower the score, the more preventive behaviors of STIs are.

**Validity And Reliability**

The content validity ratio (CVR) and content validity index (CVI) in four scales were respectively between 0.72, 1, 0.85, and 1. The impact score of all items was above 1.5. Cronbach’s alpha for each scale was as follows: perceived social fear about STIs (0.72), perceived social support about STIs (0.75), STIs Perceived risks (0.73), and perceived cognitive barriers (fear) STIs Perceived cognitive barriers(fear) about STIs (0.71).

**SECTION TWO: Demographic items**

**Please answer the following questions carefully**

1. Age …
2. Marital status
3. Permanent Marriage
4. Temporary Marriage
5. Divorced
6. The age of the first sex...
7. Education level
8. High school
9. Diploma
10. University education

5- Job

1. Housewife
2. Student
3. Employee
4. Freelancer

6- Economic situation

1. Very favorable
2. Favorable
3. Unfavorable
4. Very unfavorable

7- Have you ever had a sexually transmitted infection? Yes□ No□

8- Have you had a history of temporary marriage? Yes□ No□

**SECTION THREE: Main Variables**

a) In this section, there are questions about **STIs Perceived Social Exclusion.** Please select your answer.

| Num | Items | Completely agree | Agree | No idea | Disagree | Completely disagree |
| --- | --- | --- | --- | --- | --- | --- |
| 1 | A woman with STIs tends to avoid her acquaintances. |  |  |  |  |  |
| 2 | The spouse of a person with STIs tends to avoid sexual contact. |  |  |  |  |  |
| 3 | Friends and relatives tend to avoid a woman diagnosed with STIs. |  |  |  |  |  |
| 4 | A woman with STIs often experiences feelings of guilt. |  |  |  |  |  |
| 5 | Healthcare staff are perceived to judge individuals suffering from STIs. |  |  |  |  |  |
| 6 | A woman with STIs often feels blamed by those around her. |  |  |  |  |  |
| 7 | Society’s perception of women with STIs is generally negative. |  |  |  |  |  |

b) In this section, there are questions about **STIs Perceived Cognitive Barriers (Fear)**. Please select your answer.

| Num | Items | Completely agree | Agree | No idea | Disagree | Completely disagree |
| --- | --- | --- | --- | --- | --- | --- |
| 1 | I avoid discussing STIs due to feelings of shame and modesty. |  |  |  |  |  |
| 2 | The fear of testing positive (for AIDS, Pap smear, or female infection tests) discourages me from taking these tests. |  |  |  |  |  |
| 3 | The discomfort associated with the Pap smear test prevents me from undergoing it. |  |  |  |  |  |
| 4 | Fear associated with vaginal examinations prevents me from visiting a gynecologist or midwife. |  |  |  |  |  |
| 5 | Feelings of embarrassment over vaginal examinations deter me from visiting a gynecologist or midwife. |  |  |  |  |  |
| 6 | Indifference towards health check-ups results in me not visiting a gynecologist or midwife. |  |  |  |  |  |
| 7 | Considering gynecological visits as unimportant and a general sense of laziness prevent me from visiting a gynecologist or midwife. |  |  |  |  |  |

c) In this section, there are questions about **STIs Perceived Social Support**. Please select your answer.

| Num | Items | Completely agree | Agree | No idea | Disagree | Completely disagree |
| --- | --- | --- | --- | --- | --- | --- |
| 1 | Whenever I have a question about STIs, I ask my family members: mother or sister. |  |  |  |  |  |
| 2 | Whenever I have a question about STIs, I consult the doctor |  |  |  |  |  |
| 3 | Whenever I have a question about STIs, I consult the health care staff. |  |  |  |  |  |
| 4 | I learn some information about STIs from reputable newspapers, scientific magazines, and books. |  |  |  |  |  |
| 5 | I find some information about STIs through internet searches and virtual social networks. |  |  |  |  |  |

d) In this section, there are questions **about STIs Perceived Risks** Please select your answer.

| Num | Item | Completely agree | Agree | No idea | Disagree | Completely disagree |
| --- | --- | --- | --- | --- | --- | --- |
| 1 | I am figuring out at risk of STIs |  |  |  |  |  |
| 2 | If I being close to someone who has STIs may cause me to contract them too. |  |  |  |  |  |
| 3 | If I being close to someone who is suspected of having STIs may cause me to contract them too. |  |  |  |  |  |
| 4 | If I engaging in sexual activity with multiple partners can increase the risk of contracting sexually transmitted infections (STIs). |  |  |  |  |  |
| 5 | Some STIs, such as AIDS and hepatitis, can be fatal if I leave them untreated. |  |  |  |  |  |
| 6 | Certain STIs, such as human papillomavirus (HPV), can increase the risk of developing certain types of cancer if leave them untreated |  |  |  |  |  |
| 7 | If left untreated, sexually transmitted infections (STIs) can cause severe health complications, such as infertility. |  |  |  |  |  |
| 8 | If I use contaminated tools (needle, razor, etc..) in a beauty salon, I may contract STIs. |  |  |  |  |  |
| 9 | If I use a contaminated shared syringe, I may contract some STIs. |  |  |  |  |  |
